# Thalamocortical Mechanisms for Nostalgia-Induced Analgesia

**DOI:** 10.1523/JNEUROSCI.2123-21.2022

**Published:** 2022-04-06

**Authors:** Ming Zhang, Ziyan Yang, Jiahui Zhong, Yuqi Zhang, Xiaomin Lin, Huajian Cai, Yazhuo Kong

**Affiliations:** ^1^CAS Key Laboratory of Behavioral Science, Institute of Psychology, Chinese Academy of Sciences, Beijing 100101, China; ^2^Department of Psychology, University of Chinese Academy of Sciences, Beijing 100049, China; ^3^Research Centre of Brain and Cognitive Neuroscience, Liaoning Normal University, Dalian 116029, China; ^4^Wellcome Centre for Integrative Neuroimaging, FMRIB, Nuffield Department of Clinical Neurosciences, University of Oxford, Oxford OX3 9DU, United Kingdom

**Keywords:** analgesia, nostalgia, PAG, pain, thalamus

## Abstract

As a predominately positive emotion, nostalgia serves various adaptive functions, including a recently revealed analgesic effect. The current fMRI study aimed to explore the neural mechanisms underlying the nostalgia-induced analgesic effect on noxious thermal stimuli of different intensities. Human participants' (males and females) behavior results showed that the nostalgia paradigm significantly reduced participants' perception of pain, particularly at low pain intensities. fMRI analysis revealed that analgesia was related to decreased brain activity in pain-related brain regions, including the lingual and parahippocampal gyrus. Notably, anterior thalamic activation during the nostalgia stage predicted posterior parietal thalamus activation during the pain stage, suggesting that the thalamus might play a key role as a central functional linkage in the analgesic effect. Moreover, while thalamus-PAG functional connectivity was found to be related to nostalgic strength, periaqueductal gray-dorsolateral prefrontal cortex (PAG-dlPFC) functional connectivity was found to be associated with pain perception, suggesting possible analgesic modulatory pathways. These findings demonstrate the analgesic effect of nostalgia and, more importantly, shed light on its neural mechanism.

**SIGNIFICANCE STATEMENT** Nostalgia is known to reduce individuals' perception of physical pain. The underlying brain mechanisms, however, are unclear. Our study found that the thalamus plays a key role as a functional linkage between nostalgia and pain, suggesting a possible analgesic modulatory mechanism of nostalgia. These findings have implications for the underlying brain mechanisms of psychological analgesia.

## Introduction

Physical pain is one of the most negative physiological experiences (Hein et al., 2018). A large body of research exists on how to relieve it. Pharmacological analgesics have been established as a typical way to relieve pain; however, they are potentially addictive (Chen et al., 2014). As a result, nonpharmacological analgesics, such as electrical stimulation and acupuncture, have received increased attention (Coutaux, 2017). Numerous studies have shown that a variety of psychological treatments can manifest analgesic effects (Schwarz et al., 2016), including placebo (Eippert et al., 2009), reward acquisition (Becker et al., 2013), meditation (Zeidan and Vago, 2016), and nostalgia (Kersten et al., 2020). In the current study, we were concerned with the analgesic role of nostalgia and its underlying brain mechanism.

Nostalgia, a sentimental longing for one's past, is a self-conscious, bittersweet, but predominantly positive social emotion (Hepper et al., 2014; Sedikides et al., 2015). Nostalgia is a prevalent phenomenon triggered by various external cues, such as nostalgic music, odors, and pictures (Sedikides et al., 2015). Nostalgia is adaptive and can promote psychological well-being (Sedikides and Wildschut, 2016), improve physical comfort (Zhou et al., 2012), and reduce distress (Hussain and Alhabash, 2020). Relevant to our current study, nostalgia has been shown to relieve pain (Kersten et al., 2020). For instance, one study found that nostalgia reduced temperature-induced pain by increasing physical warmth (Zhou et al., 2012), another study found that nostalgia could help people when they experienced physical harm and made them more tolerant in a pressure algometer task (Kersten et al., 2020). However, the brain mechanism underlying the analgesic effect of nostalgia remains elusive.

Nostalgia is a complicated emotion involving self, autobiographical memory, and reward (Barrett et al., 2010; Oba et al., 2016). As a result, many brain areas relevant to these processes are implicated in nostalgia, including self-related areas such as the supramarginal gyrus (Tsakiris et al., 2007), autobiographic memory-related areas such as the hippocampus and parahippocampus, rewarding-related areas such as the ventral striatum, and emotion-related areas such as the limbic system (e.g., the amygdala and hippocampus) and the para-limbic system (e.g., the insular and frontal orbital cortex; Apaolaza-Ibantilde et al., 2010).

Pain is also implicated in broad areas of the brain, including the primary somatosensory area (SI), the secondary somatosensory area (SII; Oertel et al., 2008), the insular cortex, dorsomedial thalamus, amygdala (Panksepp, 2003), lingual gyrus (Zaki et al., 2007; Shimo et al., 2011), parahippocampal gyrus, and anterior cingulate cortex (Oertel et al., 2008). Notably, as the gateway to the cerebral cortex, the thalamus is a key relay station for transmitting nociceptive information, controlling the key to pain consciousness (Yen and Lu, 2013). Furthermore, previous connectivity analyses have found that prefrontal, parahippocampal, and brainstem structures are involved in the modulation of emotion when experiencing pain (Roy et al., 2009), suggesting that nostalgia may modulate pain via these top-down pathways.

In the current investigation, we examined whether there would be an analgesic effect of nostalgia under various pain intensities and, if so, what the underlying brain mechanism could be. The experimental paradigm included a nostalgic picture (vs a control one) to induce nostalgia sessions followed by pain sessions with low-intensity and high-intensity nociceptive thermal stimuli. Although the existing nostalgia-related neuroimaging research did not allow us to make an exact hypothesis, some tentative expectations could be derived from the role of the thalamocortical system in modulating pain (Qin et al., 2020). Specifically, after experiencing nostalgia, the thalamocortical system might integrate outside signals (i.e., nostalgic information) into the current mental state (i.e., pain perception; Shih et al., 2019); and then, nostalgic analgesia might be induced by top-down modulation from the well-known pain descending modulatory regions, such as the brainstem (Oliva et al., 2021).

## Materials and Methods

### Participants

A priori power analysis demonstrated that a sample size of 34 would allow for the detection of an effect size (*f* = 0.25) with 80% power at an α of 0.05 for the repeated measures with two within-participant factors (Kersten et al., 2020). A total of 34 right-handed participants (18 females, age = 21.50 ± 2.05 years, range = 18–25 years) took part in this study. Participants were screened before taking part in the study using the Pain Sensitivity Scale (PSS; e.g., “Imagine you burn your tongue on a very hot drink”; responses were rated on a scale from 1 = “no pain” to 10 = “pain as bad as it could be”; Ruscheweyh et al., 2009; Quan et al., 2018) and the Southampton Nostalgia Scale (SNS; e.g., “How valuable is nostalgia for you?”; responses were rated on a scale from 1 = “not at all” to 7 = “very much”; Routledge et al., 2008; Barrett et al., 2010). Participants with a mean PSS score ≥ 3.1 (4.7 ± 1.6 means PSQ-moderate, Ruscheweyh et al., 2009) and a mean SNS score ≥4 (<4 means low nostalgia inclination; Sugimori et al., 2020) were selected to increase the chance that the experimental manipulation would be effective. The selected participants had no neurologic or psychiatric history. They were instructed not to ingest any alcohol or pain medicine for at least 4 h before participating in the experiment (Mercer and Holder, 1997; Kanarek and Carrington, 2004). They completed a thorough written and verbally informed consent process after arriving at the lab. Before entering the MRI scanner, they completed a magnetic resonance imaging research center questionnaire that required all individuals to report their current health status and medical records, including physical injuries and mental disorders. All participants were fully debriefed and received RMB 150 as compensation for participating in the study. The experimental procedures were approved by the Institutional Review Board of the Institute of Psychology at the Chinese Academy of Sciences and were performed in accordance with the Helsinki Declaration.

### Materials

**Figure 1.**
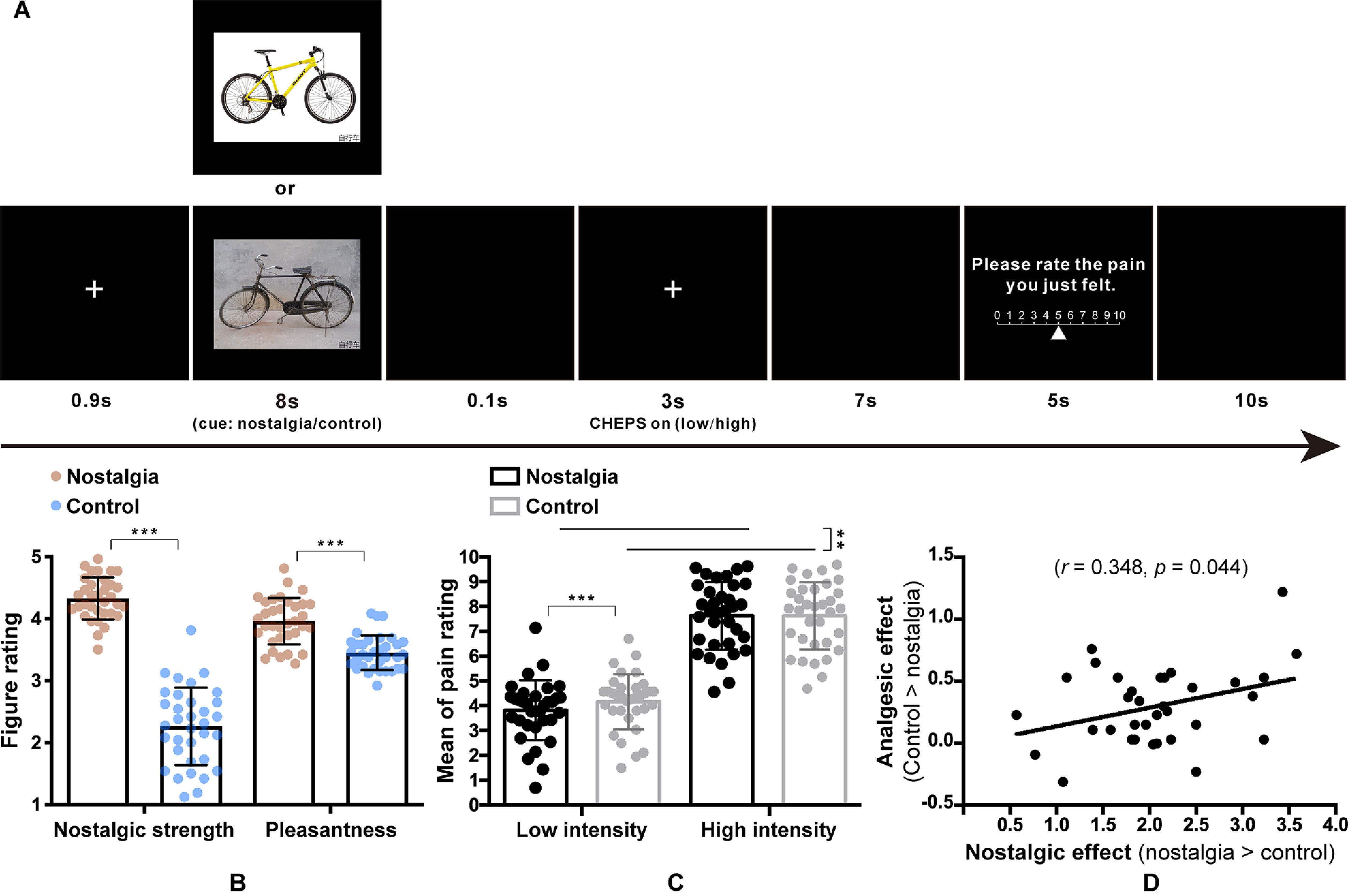
***A***, The setup for each trial. In the current trial, participants viewed a nostalgic cue (i.e., a bicycle from childhood), while in the control trial, participants viewed a control cue (i.e., a bicycle from contemporary life). ***B***, Manipulation check performed using a five-point Likert scale (error bars represent SD, ***p < 0.001). ***C***, Mean of pain ratings in the four conditions (**p < 0.01). ***D***, Correlation between the nostalgic effect and analgesic effect.

The study used 26 nostalgic images and 26 control images (see Fig. 1 for material samples) that were successfully used to induce nostalgic feelings in a previous study (for more details, see Yang et al., 2021). The nostalgic pictures depicted objects or scenes from childhood, whereas the control pictures depicted corresponding objects or scenes from modern life. In the current study, the visual stimuli (visual angle 11.18° × 10.20°) were presented on a uniform black background and displayed via a video projector (frequency 60 Hz, resolution 1920 × 1080) onto a rear-projection screen mounted at the head of the scanner bore. Participants viewed the stimuli through a mirror on a head coil positioned over their eyes.

### Thermal pain stimuli

All thermal pain stimuli were produced by a Medoc 9-cm^2^ contact heat-evoked potential stimulator (CHEPS). In the scanner, the heat pain threshold was assessed first to define the low and high intensities (i.e., threshold temperature plus 1°C vs 3°C, i.e., 43.35 ± 1.67°C vs 45.35 ± 1.67°C; Dellapina et al., 2011; Tabry et al., 2020). The heat pain threshold was assessed on the right forearm, 10 cm above the wrist, with a 3-s interstimulus interval and a 40°C/s rate of temperature rise. Participants reported the pain they experienced for the brief thermal stimuli using a numerical pain rating scale ranging from 0 to 10 (0 = no feeling, 1 = a feeling of warmth, 2 = a feeling of heat, 3 = a feeling of hotness, 4 = just a feeling of pain, 10 = a feeling of pain as bad as it could be. Values from 4 to 10 gradually increased the degree of pain; Hu et al., 2014; Hu and Iannetti, 2019; Zhang et al., 2021). The mean intensity that participants reported as the point where they first began to feel pain (i.e., number 4) three times over was used as the threshold temperature. In the experiment, the pain ratings of the thermal stimuli were measured based on the subjects' responses to 52 heat pulses at either the lower or higher intensities.

### Procedure

Stimulus presentation and behavioral response collection were controlled by E-Prime 2.0 (Psychological Software Tools). Participants performed a practice experiment outside the MRI scanner using the same procedure as in the actual experiment. There were 52 trials performed for the conditions (nostalgia vs control) and intensities (low vs high) for a total of three sessions. Participants were instructed to view these pictures carefully before starting each session. The trial sequence in each session was pseudo-randomized with a trial time of 34 s. Each trial proceeded as follows (see Fig. 1*A*). First, a white fixation cross was presented for 0.9 s, and then one of the two cues (nostalgia or control) was presented for 8 s. Subsequently, a white fixation cross was presented for 0.1 s; at the same time, a heat pulse (low or high) was delivered to the right forearm (for 3 s). A white fixation cross was then presented for 7 s. After that, participants were asked to perceive the pain they just felt and to provide pain ratings for the brief thermal stimuli using the numerical pain rating scale (displayed for 5 s) ranging from 0 (“no pain”) to 10 (“pain as bad as it could be”), with four denoting the threshold of pain, using their left hand on a response box. Subsequently, a black background screen appeared for 10 s before the next trial began.

Finally, outside the MRI machine, a manipulation check was performed with participants being asked to rate the nostalgic strength of each picture (“To what extent does this picture make you feel nostalgic?” Responses were rated from 1 = “not at all” to 5 = “very much”). To examine the pleasantness of the nostalgia pictures, we also asked participants to rate each picture (“To what extent does this picture make you feel pleasant?”; responses were rated from 1 = “very unpleasant” to 5 = “very pleasant”; Oba et al., 2016).

### Data acquisition

A GE Discovery MR750 3T scanner (GE Medical Systems) in combination with an 8-channel head matrix coil was used for functional brain imaging in the present study. The participant's head was securely but comfortably stabilized with firm foam padding. Functional data were acquired using an echoplanar imaging (EPI) sequence using an axial slice orientation (37 slices, TR/TE = 2000/30 ms, slice thickness = 3.5 mm, FOV = 224 mm, flip angle = 90°, matrix size: 64 × 64) covering the whole brain. A high-resolution T1-weighted 3D SPGR sequence was acquired between the first and second fMRI sessions (192 slices, TR/TE = 6.7/Min Full ms, slice thickness = 1.0 mm, FOV = 256 mm, flip angle = 12°, matrix = 256 × 256).

### Data analysis

Data were analyzed using the FEAT (FMRI Expert Analysis Tool) version 6.00, part of FSL (FMRIB's Software Library; https://www.fmrib.ox.ac.uk/fsl). At the individual level, the following preprocessing steps were applied: motion correction using MCFLIRT (Jenkinson et al., 2002), nonbrain removal using BET (Smith, 2002), spatial smoothing using a Gaussian kernel of full-width at half-maximum (FWHM) 5 mm, grand-mean intensity normalization of the entire 4D dataset by a single multiplicative factor, and high-pass temporal filtering. Registration from functional images to high-resolution structures was conducted using FLIRT (Jenkinson and Smith, 2001; Jenkinson et al., 2002). Registration from a high-resolution structure to a standard space was further refined using FNIRT nonlinear registration (Andersson et al., 2007a, b). Each session of fMRI data were modeled on a voxel-by-voxel basis using a general linear model (GLM) approach (Woolrich et al., 2001), and parameter estimates (PEs) were estimated for nostalgia or control cue stages, followed by low/high pain stimuli. A second-level analysis of the fixed-effects model was performed on within-subject activation across the three sessions. Finally, the group level analysis was conducted using a mixed-effects approach (FLAME, FMRIB's Local Analysis of Mixed Effects; Beckmann et al., 2003; Woolrich et al., 2004; Woolrich, 2008), and *Z* (Gaussianized T/F) statistic images were thresholded using clusters determined by *Z* > 2.3 and a corrected cluster significance threshold of *p* = 0.05 (Worsley, 2001). A repeated measure ANOVA (Schestatsky et al., 2008) and the independent sample *t* test were performed across subjects to investigate the brain regions involved in the variability of responses at low or high pain intensities under the nostalgia or control condition (i.e., four combined conditions: nostalgia-low, control-low, nostalgia-high, and control-high).

Brain regions with significantly contrasting activation differences (control > nostalgia) in the pain stage were flagged for a region of interest (ROI) analysis (Cozzolino et al., 2019). Masks of ROIs were created in FSLeyes (part of FSL tools, https://fsl.fmrib.ox.ac.uk/fsl/fsleyes/) and further thresholded using Harvard-Oxford cortical and subcortical atlases. The average PE values within ROIs (including the lingual gyrus and parahippocampal gyrus) were extracted from the four conditions for further analysis.

To explore the mechanism of nostalgia-induced analgesia, a GLM was used with the nostalgic strength (i.e., the nostalgic rating of figures) and the analgesic effect (i.e., the difference in the pain rating in the control condition compared with the nostalgia condition in the pain stage) as regressors of interest to determine the nostalgia and pain encoding brain activation across the whole brain. Statistical images for encoding activation were thresholded using a cluster-forming correction determined by *Z* > 2.3 and a corrected cluster significance threshold of *p* < 0.05.

Finally, brain regions that were significantly correlated to nostalgic strength were further taken to the ROI masks (the prefrontal thalamus) for further psychophysiological interaction (PPI) analysis in the cue stage. We first extracted the mean time course from the prefrontal thalamus seed region using preprocessed functional data. Next, the time course was added to the GLM at the individual level as the physiological regressor, with the original task regressors as the psychological regressors. The final interaction regressor is the scalar product of the psychological and physiological regressors. Individual PEs for psychophysiological interaction (PPI) were then taken to the normal higher-level group comparison (PPI analysis in Feat; https://fsl.fmrib.ox.ac.uk/fsl/fslwiki/PPIHowToRun). We also performed further PPI analyses during the pain stage. The PAG (adopted from Harvard-Oxford cortical and subcortical atlases) was used for seed voxel identification because it correlated with nostalgic strength in the cue stage, which allowed us to test whether there was functional connectivity that was associated with the pain ratings.

## Results

### Behavioral results

#### Postexperiment manipulation check

As intended, the independent sample *t* tests revealed that participants felt more nostalgia toward the nostalgic pictures (mean ± SD, 4.32 ± 0.34) than toward the control pictures (2.26 ± 0.62, *t*_(66)_ = 16.92, *p* < 0.001, *d* = 4.12), indicating that the manipulation worked. Also, participants felt more pleasant toward the nostalgic pictures (3.96 ± 0.37) than toward the control pictures (3.45 ± 0.28, *t*_(66)_ = 6.35, *p* < 0.001, *d* = 1.55; Fig. 1*B*), suggesting that nostalgia was overall a positive emotion. A regression analysis with experimental conditions and pleasantness as predictors (*R*^2^ = 0.912) also showed that aroused pleasantness was positively associated with aroused nostalgia (β = 0.478, *p* = 0.010).

#### Effect of nostalgia on pain ratings

Pain ratings were analyzed by two-way repeated ANOVAs with condition and intensity as two within-participant variables. The main effect of nostalgia condition in the pain stage was significant, *F*_(1,33)_ = 10.71, *p* = 0.003, η*_p_^2^* = 0.245, indicating that, as demonstrated in a previous study (Kersten et al., 2020), nostalgia significantly reduced pain ratings. The main effect of pain intensity was also significant, *F*_(1,33)_ = 227.53, *p* < 0.001, η*_p_^2^* = 0.873, suggesting that stronger pain stimuli led to a stronger pain rating. The interaction between condition and intensity was significant, *F*_(1,33)_ = 14.10, *p* = 0.001, η*_p_^2^* = 0.299. *Post hoc* analysis showed that the pain rating in the nostalgia condition (3.82 ± 1.21) was significantly lower than that in the control condition (4.16 ± 1.12) at the low pain intensity level, *t*_(33)_ = –4.42, *p* < 0.001, *d* = 0.29 (Fig. 1*C*); however, there was no significant difference between these two conditions at the high pain intensity level (7.62 ± 1.36 vs 7.62 ± 1.35, *t*_(33)_ ≈ 0, *p* = 0.998).

#### Correlation between nostalgic and analgesic effects

We calculated an index of relative nostalgic strength (i.e., the nostalgic effect, the nostalgic ratings of the nostalgic pictures minus those of the control ones), with a larger number suggesting a stronger nostalgic effect. We also calculated an index of the analgesic effect by subtracting the pain rating in the control condition from that in the paired nostalgia condition in the pain stage, with a larger number denoting a stronger analgesic effect. We then examined the correlation between nostalgic and analgesic effects. The results revealed a positive correlation (Fig. 1*D*; *r* = 0.348, *p* = 0.044, *p_(corr_fdr)_* = 0.0873; corrected for multiple comparisons based on the more stringent false discovery rate proposed by Fachada and Rosa, 2018), suggesting that stronger nostalgia was associated with a large analgesic effect; that is, the more nostalgic the participants felt, the less pain they perceived. As for the pleasantness effect (i.e., the relative pleasantness strength, the pleasantness ratings of the nostalgic pictures minus those of the control ones), we found that it was not significantly correlated with the analgesic effect (*r* = 0.002, *p* = 0.993). This suggests that the analgesic effect should be elicited by nostalgia rather than from the pleasantness induced by the pictures.

### fMRI results

#### Whole-brain ANOVA

**Figure 2.**
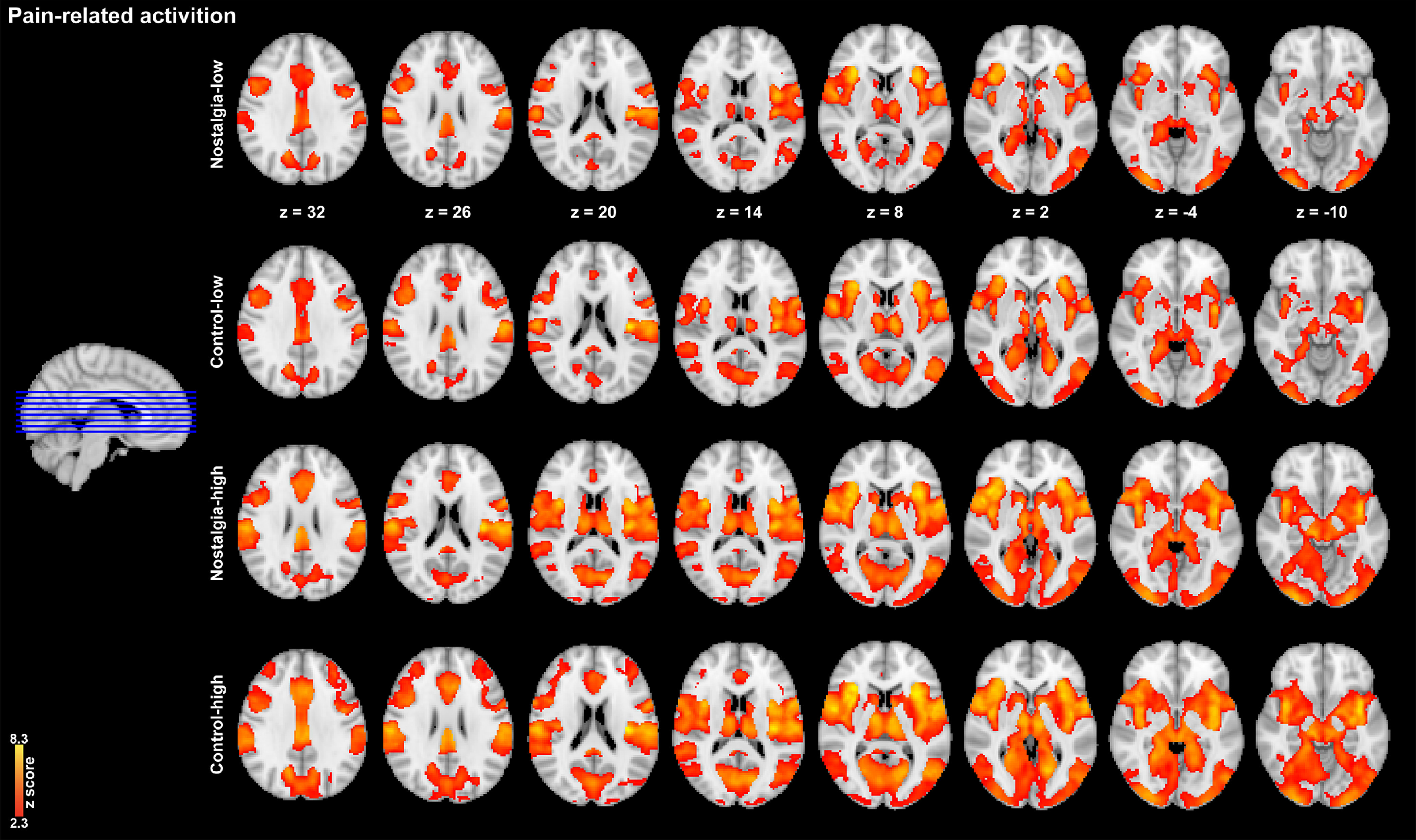
Pain-related activation in four conditions.

**Figure 3.**
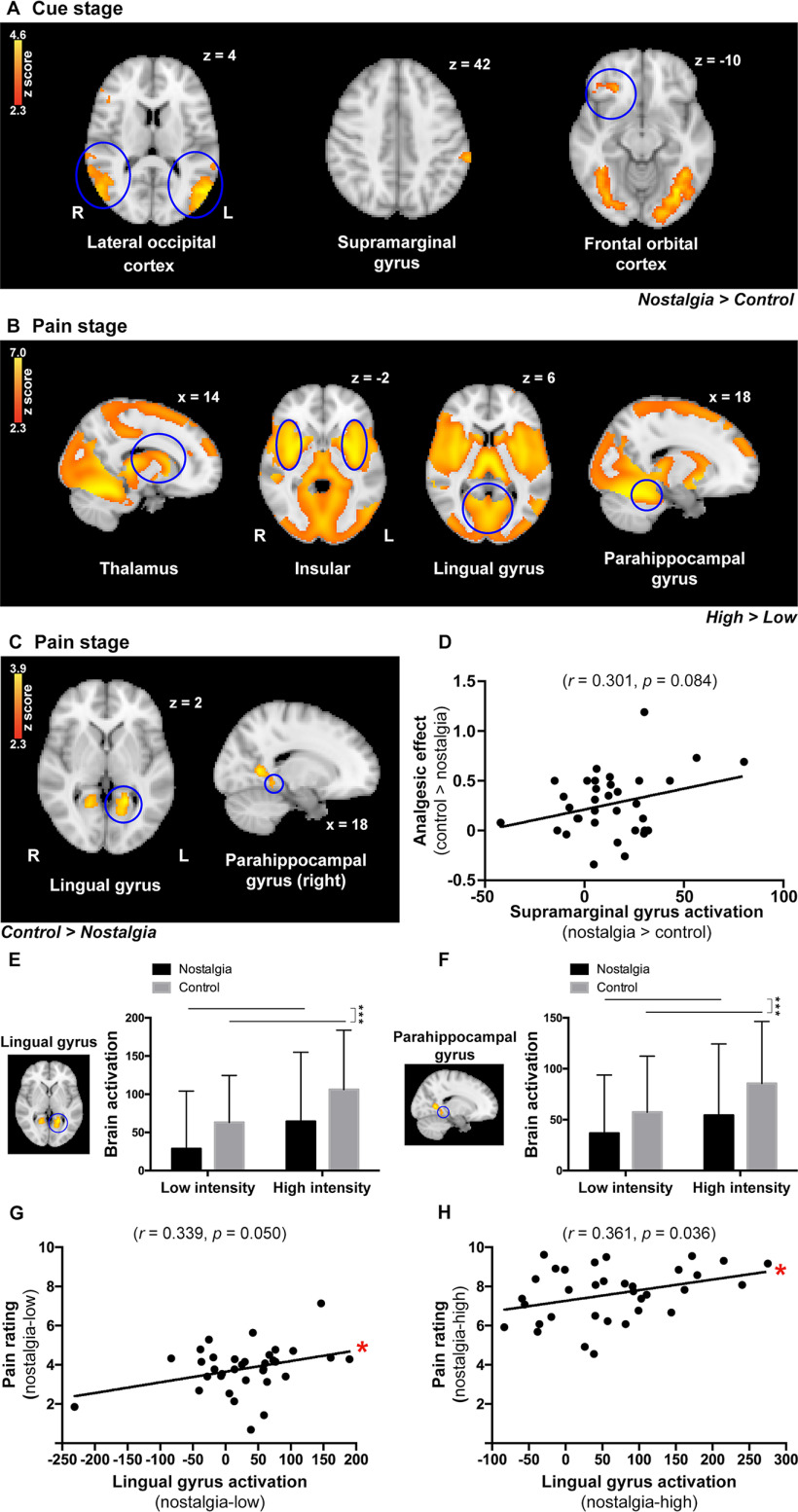
***A***, During the cue stage, brain activation of the lateral occipital cortex, the left supramarginal gyrus, and the right frontal orbital cortex was significantly increased in the nostalgia condition compared with the control condition. ***B***, During the pain stage, brain activation of the thalamus, insular, lingual gyrus, and parahippocampal gyrus was significantly increased in the high-intensity condition compared with the low-intensity condition. ***C***, Brain activation of the lingual gyrus and parahippocampal gyrus was significantly greater in the control condition compared with the nostalgia condition in the pain stage. ***D***, Correlation between supramarginal gyrus activation (nostalgia > control) and the analgesic effect (control > nostalgia). ***E***, ROI analysis revealed that brain activation of the lingual gyrus was significantly lower in the nostalgia condition compared with the control condition. ***F***, ROI analysis revealed that brain activation of the parahippocampal gyrus was significantly lower in the nostalgia condition compared with the control condition. ***G***, Correlation between lingual gyrus activation and pain rating in the nostalgia-low condition. ***H***, Correlation between lingual gyrus activation and pain rating in the nostalgia-high condition (*p ≤ 0.05, ***p < 0.001).

Consistent with the results of the previous studies, the classic pain-related regions (i.e., the SI, SII, insular) evoked by the thermal noxious stimuli were observed in every single condition (i.e., nostalgia/control, low/high pain levels; Fig. 2). In the cue stage, the analysis of the fMRI results revealed nostalgia-specific activation in the lateral occipital cortex ([50, –66, 4], [–52, –68, 12]), the left supramarginal gyrus ([–64, –34, 42]), and the right frontal orbital cortex ([26, 32, –10]) in the nostalgia condition compared with the control condition (Fig. 3*A*). Brain activation of supramarginal gyrus (nostalgia > control) was marginally positively correlated with the analgesic effect (control > nostalgia; *r* = 0.301, *p* = 0.084; Fig. 3*D*). We also checked the deactivation in the nostalgia condition in contrast to the control condition. We found only two deactivated regions, the cingulate gyrus ([0, –22, 36]) and angular gyrus ([–50, –54, 36]).

In the pain stage, the bilateral SI ([–46, –24, 46], [48, –18, 46]), SII ([–54, –30, 20], [54, –24, 20]), thalamus ([–16, –30, 8], [14, –18, 10]), insular ([–42, –2, –2], [38, –2, –2]), lingual gyrus ([–22, –56, 2], [18, –56, 6]), and parahippocampal gyrus ([–12, –42, –8], [18, –42, –8]) were increased in the high-intensity condition than in the low-intensity condition (Fig. 3*B*). The increased activation (high > low) was positively correlated with increased pain ratings (high > low; *r*_SI_ = 0.381, *p* = 0.026, *p_(corr_fdr)_* = 0.0522; *r*_SII_ = 0.421, *p* = 0.013, *p_(corr_fdr)_* = 0.0265; *r*_thalamus_ = 0.509, *p* = 0.002, *p_(corr_fdr)_* = 0.0042; *r*_insular_ = 0.449, *p* = 0.008, *p_(corr_fdr)_* = 0.0154; *r*_lingual gyrus_ = 0.401, *p* = 0.019, *p_(corr_fdr)_* = 0.0376), except for the parahippocampal gyrus (*r*_parahippocampal gyrus_ = 0.257, *p* = 0.142), implying that the stronger the thermal stimulus, the stronger the activation of the pain-related brain area. Importantly, greater activation was visible in the bilateral lingual gyrus ([–18, –52, 2], [18, –56, 6]) and the right parahippocampal gyrus ([18, –42, –8]) in the control condition than in the nostalgia condition (Fig. 3*C*).

#### ROI analysis

We examined the relationship between nostalgic strength and activation in pain-related neural regions. Based on the results of the whole-brain analyses, we focused on the ROIs in the lingual and parahippocampal gyri. As expected, participants showed decreased activation in the lingual gyrus and parahippocampal gyrus in the nostalgia condition compared with the control condition in the pain stage [*t*_(66)_ = –4.17, *p* < 0.001, *d* = 0.47 (Fig. 3*E*); *t*_(66)_ = –3.98, *p* < 0.001, *d* = 0.44 (Fig. 3*F*)]. We then examined the correlations between pain ratings and brain activation at each of the four pain conditions (i.e., nostalgia-high, nostalgia-low, control-high, and control-low). For the lingual gyrus, pain ratings were positively correlated with brain activity in the nostalgia-low and nostalgia-high conditions [*r* = 0.339, *p* = 0.050, *p_(corr_fdr)_* = 0.0992 (Fig. 3*G*); *r* = 0.361, *p* = 0.036, *p_(corr_fdr)_* = 0.0723 (Fig. 3*H*)], but not in the control-low and control-high conditions (*r* = 0.230, *p* = 0.192; *r* = 0.326, *p* = 0.060), suggesting that nostalgia played a key role in pain-related activation in the lingual gyrus, regardless of the intensity of the pain stimuli. For the parahippocampal gyrus, no significant correlations were found (*r* = 0.318, *p* = 0.067; *r* = 0.212, *p* = 0.228; *r* = 0.109, *p* = 0.540), except in the control-high condition (*r* = 0.397, *p* = 0.02, *p_(corr_fdr)_* = 0.04).

#### Nostalgia and pain encoding activities

**Figure 4.**
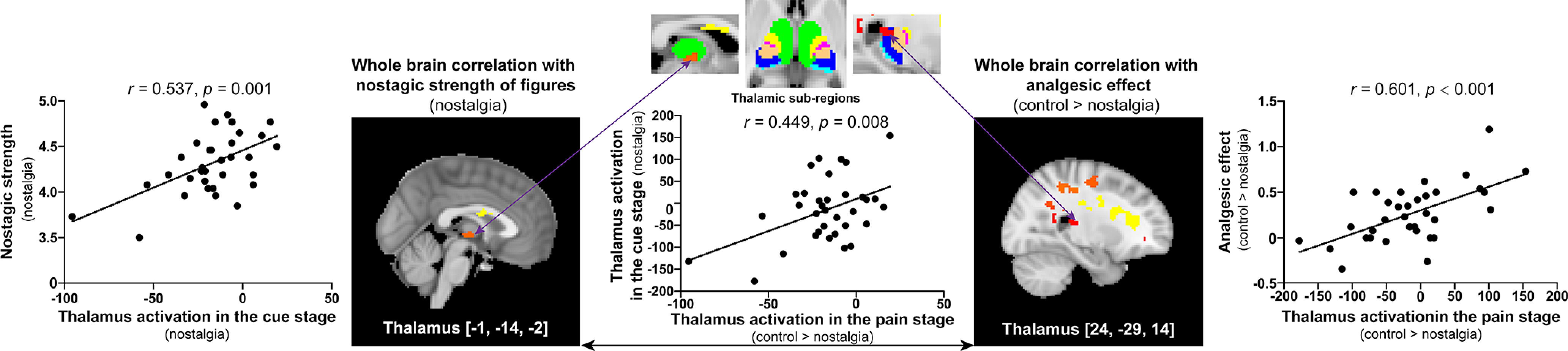
Significant correlations between brain activation and behavioral scores in the cue and pain stages. Left, During nostalgia encoding, the prefrontal thalamus [–1, –14, –2] showed a positive correlation between the BOLD response magnitude and nostalgic strength. Middle, Brain activity in the prefrontal thalamus in the cue stage was positively correlated to brain activity in the posterior parietal thalamus in the pain stage. Right, During pain encoding, the posterior parietal thalamus [24, –29, 14] showed a positive correlation between the BOLD response and the analgesic effect.

Nostalgic strength was positively correlated with brain activity in the prefrontal thalamus ([–1, –14, –2]) in the nostalgia stage (*r* = 0.537, *p* = 0.001, *p_(corr_fdr)_* = 0.0021; Fig. 4, left part); in addition, analgesic effects were positively correlated with brain activity in the posterior parietal thalamus ([24, –29, 14]) during the pain stage (*r* = 0.601, *p* < 0.001, *p_(corr_fdr)_* < 0.001; Fig. 4, right part). These findings suggest that nostalgia could affect thalamic activity not only during the nostalgia stage but also during the pain stage. Moreover, brain activity in the thalamus in the nostalgia ([–1, –14, –2]) and pain stages ([24, –29, 14]) were positively correlated with each other (*r* = 0.449, *p* = 0.008, *p_(corr_fdr)_* = 0.0155; Fig. 4, middle part).

**Figure 5.**
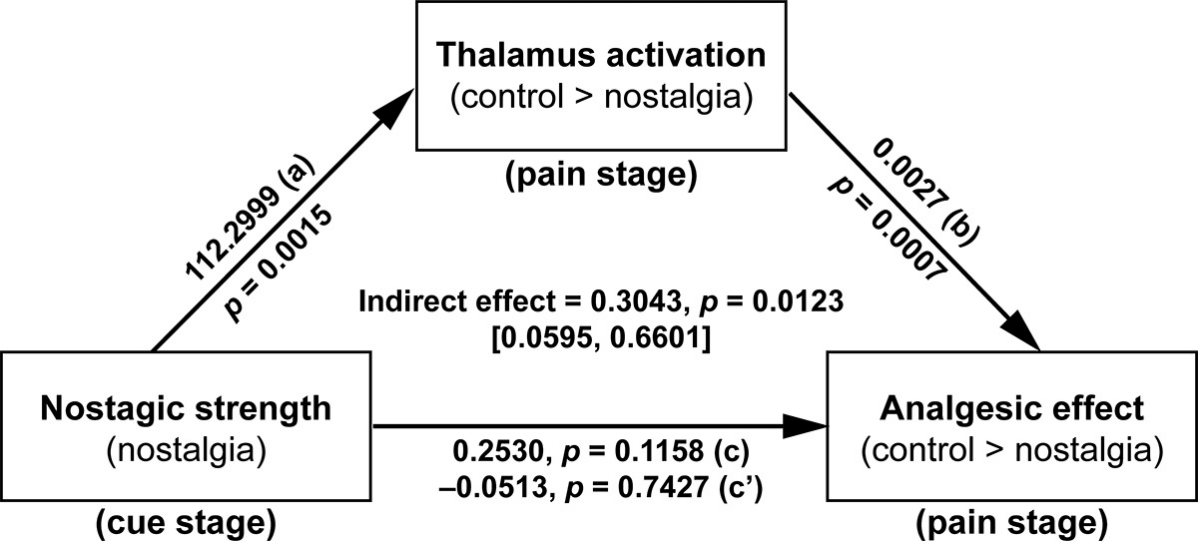
Thalamus activation mediated how nostalgia affected the analgesic effect.

These findings suggest that the thalamus might play a key role in the nostalgia and pain information encoding process in the possible brain circuit for nostalgia-induced analgesia, which we tested using mediation analysis. Overall, the model with brain activation (in the posterior parietal thalamus) in the pain stage as the mediator was significant (*R*^2^ = 0.36, MSE = 0.07, *F*_(2,31)_ = 8.85, *p* = 0.0009). The indirect effect via activation in the pain stage was significant (*b* = 0.30, SE = 0.16, 95%CI = [0.06, 0.66]; Fig. 5), thus confirming our expectation that activity in the thalamus plays a regulatory role in generating an analgesic effect.

#### PPI analysis

**Figure 6.**
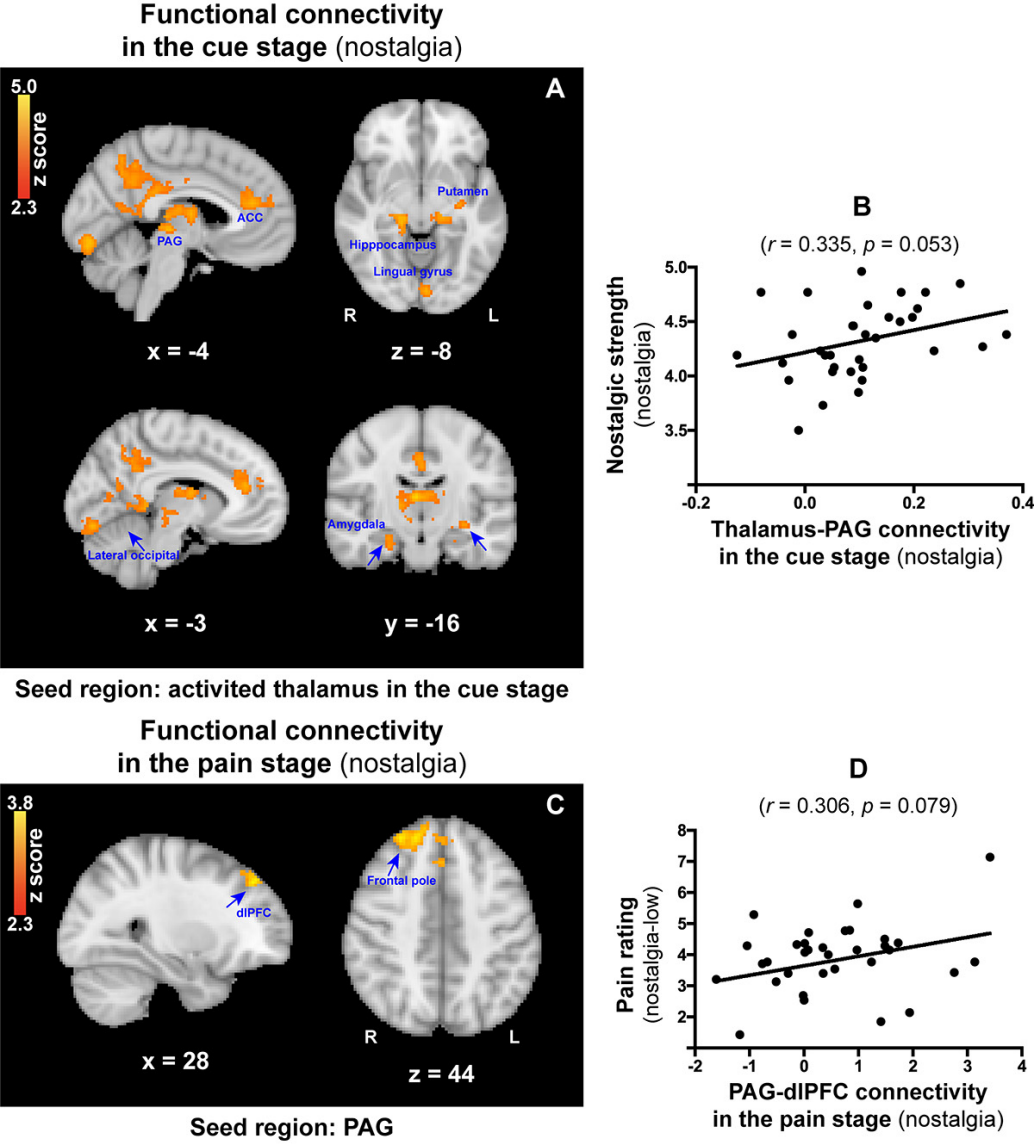
***A***, Functional connectivity between the BOLD time-series signals in the prefrontal thalamus (seed region) and PAG, as well as in the putamen, amygdala, and hippocampus. ***B***, Correlation between the thalamus-PAG connectivity in the cue stage and nostalgic strength in the nostalgia condition. ***C***, Functional connectivity between the BOLD time-series signals in the PAG (seed region), dlPFC, and the frontal pole in the pain stage. ***D***, Correlation between the PAG-dlPFC connectivity in the pain stage and the pain rating in the nostalgia-low condition.

Whole-brain PPI analysis revealed strong functional connectivity between the thalamus (seed region) and PAG ([–4, –27, –3]), as well as with several other regions, including the putamen ([–32, –15, –8]), amygdala ([24, –17, –16]), and hippocampus ([16, –27, –8]; Fig. 6*A*) during the nostalgia stage. Notably, thalamus-PAG connectivity was positively correlated with nostalgic strength in the cue stage (*r* = 0.335, *p* = 0.053, *p_(corr_fdr)_* = 0.1051; Fig. 6*B*), which indicates that the greater the nostalgic strength, the stronger the connection between the thalamus and the PAG. Another whole-brain PPI analysis with the PAG as the seed was conducted with regard to the pain stage. It revealed significant functional connectivity between the PAG and dlPFC ([28, 40, 44]), as well as with the frontal pole ([19, 47, 44]; Fig. 6*C*) in the nostalgia condition. In contrast, no significant functional connectivity was observed in the control condition. Meanwhile, PAG-dlPFC connectivity was marginally positively correlated with the pain rating in the nostalgia-low condition (*r* = 0.306, *p* = 0.079; Fig. 6*D*), reflecting a modulation associated with the pain rating of low-intensity noxious stimuli.

## Discussion

In this study, we examined the neural mechanisms underlying the analgesic effect of nostalgia. Similar to the findings of previous behavioral studies (Zhou et al., 2012; Kersten et al., 2020), we observed a direct analgesic effect of nostalgia on pain, particularly for low-intensity pain. Further, based on behavioral evidence, we found that nostalgia significantly attenuated brain responses to thermal pain in the lingual gyrus and parahippocampal gyrus in comparison to the control condition. Most importantly, the thalamocortical system was proven to play a vital role in analgesia. First, the thalamus was highly engaged in both nostalgia and pain encoding processes. Second, thalamic activity in the pain stage was shown to mediate the effect of nostalgic strength on pain. Third, nostalgic strength was highly associated with thalamus-PAG connectivity in the cue stage, and PAG-dlPFC coupling predicted pain perception in the following pain stage, both of which are important pathways in analgesia modulation. Overall, we demonstrated the analgesic effect of nostalgia and elucidated its neural mechanism.

### Nostalgia-induced analgesic effects

As a positive emotion, nostalgia can help maintain positive psychological status and counteract negative situations (Wildschut et al., 2006), such as painful experiences (Zhou et al., 2012). Notably, the current study found that, after being shown nostalgic stimuli (vs non-nostalgia or control stimuli), participants reported significantly weaker feeling of pain, which was not the case for those shown non-nostalgic stimuli. We also found that the analgesic effect was positively correlated with the nostalgic effect (Fig. 1*D*).

Nostalgia-induced analgesic effects were confirmed in our experiment; however, this effect was only significant for relatively weak noxious stimuli. A possible reason for this is that the effect of nostalgic cues could last longer when the pain intensity is low. Another possible reason may be that severe pain itself occupies more cognitive resources and therefore weakens the effect of nostalgia cues (Levine et al., 1979). These results suggest that nostalgia would be more effective for mild clinical pain.

### Brain activation involved in nostalgia

Our study found nostalgia-specific activation in the lateral occipital cortex, supramarginal gyrus, and frontal orbital cortex. These regions are all involved in retro scene processing (Yücel et al., 2020), the sensation of the self (Tsakiris et al., 2007), and emotional appraisal (Rolls, 2004; Sotres-Bayon et al., 2006). Compared with the effect of recalling a nostalgic experience, observing nostalgic stimuli, as was done in the current study, might not be strong enough to arouse activity in the reward-related regions of the brain (Barrett and Janata, 2016; Oba et al., 2016). However, the self-related and emotion-related regions are evoked by the stimuli, which also play an important role in nostalgia processing (Tsakiris et al., 2007; Apaolaza-Ibantilde et al., 2010).

### Brain activation involved in pain under nostalgic effects

Interestingly, brain activation of the left lingual gyrus and parahippocampal gyrus decreased significantly in the nostalgia condition, showing a common modulation effect induced by nostalgia. As discussed above, nostalgic cues tend to elicit a positive psychological status despite perceiving noxious stimuli (Kersten et al., 2020). Meanwhile, the lingual gyrus is associated with the emotional regulation of autobiographical memories (Kross et al., 2009; Rubin-Falcone et al., 2018). After participants perceived the positive nostalgic information, the inhibited brain activation evoked by thermal stimulus reflected both self-related and emotion-related modulation.

In this study, we did not find a significant discrepancy in other classic pain-related regions (e.g., the insular) in the nostalgia condition compared with the control condition using the ANOVA within the routine GLM. The nostalgia-/pain-specific activated regions in the initial whole-brain GLM analysis were not the same as those found in the nostalgia/pain encoding activities. This suggests that nostalgia perception/encoding, pain perception/encoding, and analgesia regulation might implicate different pathways or mechanisms. Our study was more concerned about how nostalgia may produce analgesic effects by modulating the pain perception encoding process. As a result, we mainly focused on the role of the thalamus in examining how the underlying nostalgia-induced analgesia functions, which will be discussed below.

### Thalamocortical mechanisms involved in nostalgia and analgesia encoding

In our study, the thalamus was associated with both nostalgia and analgesia encoding. The thalamus is an important brain region for information transmission, integration, and pain modulation (Ploner et al., 2010). Specifically, the nostalgia-encoding region within the prefrontal thalamus is the thalamic subregion connected to the prefrontal lobe (Garibotto et al., 2020; Culbreth et al., 2021), which is known to be critical for higher cognitive functions (Mitchell, 2015). In contrast, analgesia encoding within the posterior parietal thalamus involves the thalamic subregion connected to the posterior parietal lobe (Liu et al., 2019; Garibotto et al., 2020), contributing to multisensory and sensory-motor integration (Gilissen et al., 2021). More importantly, brain activity in the prefrontal and posterior parietal thalamus, which separately encode nostalgia and analgesia, were significantly positively correlated in the current study. Additionally, mediation analysis found that nostalgia may attenuate pain by strengthening the activity of the thalamus during the pain stage (Fig. 5). The thalamus integrates the information generated by the nostalgic state (Krause et al., 2019), implying a thalamus-based central functional linkage in the nostalgia-induced analgesic process.

In addition to the mediating role of the thalamus, we also found that thalamus-PAG connectivity was positively correlated with nostalgic strength, and that PAG-dlPFC connectivity was salient in response to nostalgic stimuli and correlated with pain ratings in the nostalgic-low condition. It is well understood that the PAG plays a crucial role in the descending pain inhibition system (Dougherty et al., 2008; Grahl et al., 2018) and is associated with analgesia (Yilmaz et al., 2010). A previous study has shown that thalamus-PAG connectivity predicts a greater analgesic effect after sham and real tDCS (Cummiford et al., 2016). It is possible that these effects of prestimulus connectivity related to nostalgia between the thalamus and PAG might remain active for subsequent noxious stimuli.

It has also been reported that PAG-dlPFC functional connectivity is associated with a placebo analgesic response (Wager et al., 2004). Our results also showed that PAG-dlPFC functional connectivity was related to pain ratings in the nostalgia-low condition. The dlPFC is engaged in cognitive-affective processing of pain, and it has been suggested that it exerts an active control on pain perception by top-down modulation (Weizman et al., 2018; Mao et al., 2020). In consideration of this, we interpret the current study results as indicating that nostalgic analgesia was more effective at the low-intensity level, which had been observed in behavioral performance.

**Figure 7.**
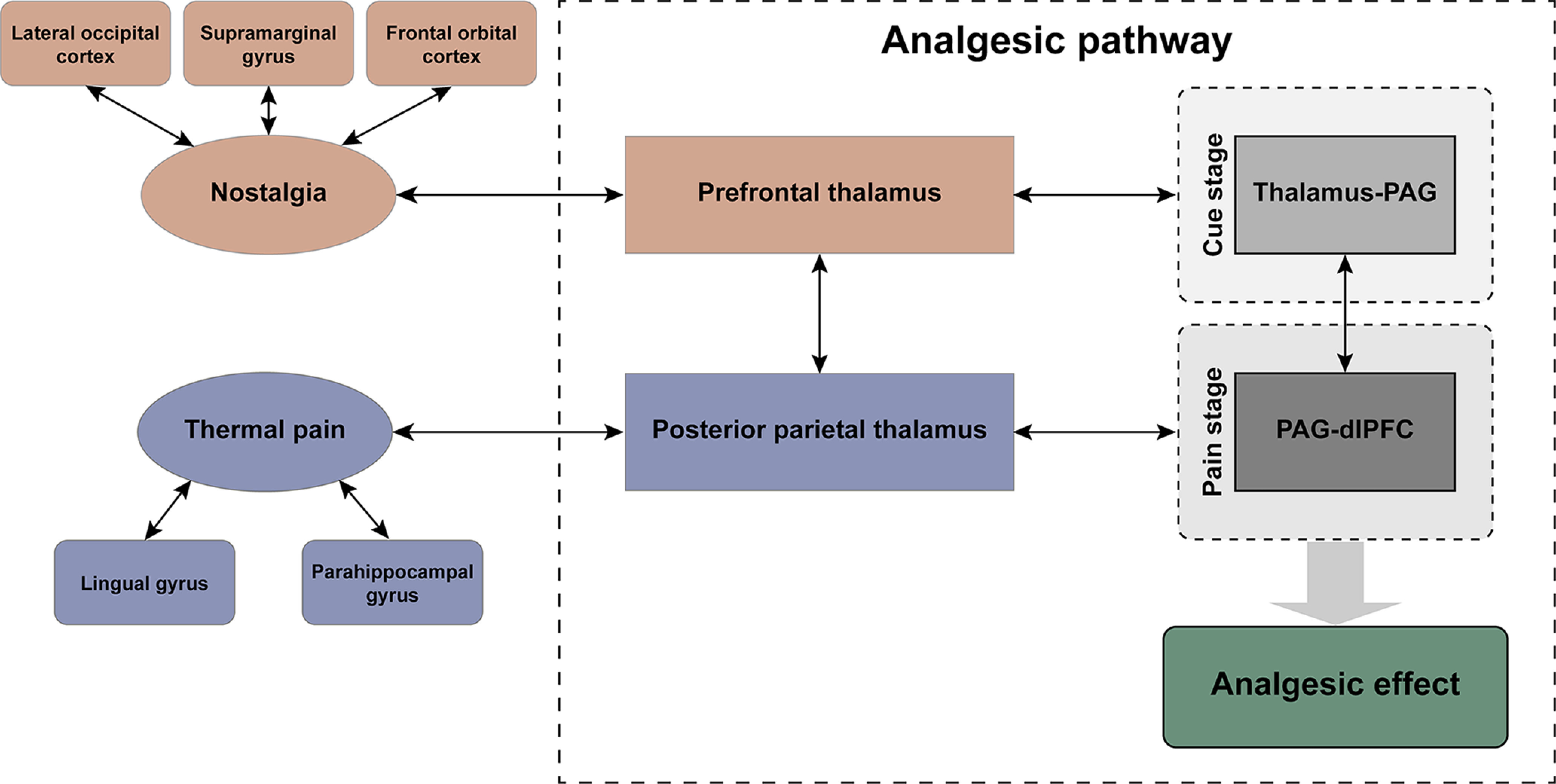
The model of thalamus-centered pathways affected by the analgesic effect associated with nostalgia.

Based on our findings, we propose a possible model of thalamus-centered pathways to explain the analgesic effect of nostalgia (Fig. 7). The thalamus modulates nociceptive inputs and plays a crucial role in triggering the brain stem analgesic pathway. We speculate that the thalamus integrates information under the effect of nostalgia and transmits downstream signals to the PAG. The PAG then transmits the regulatory signal back to the dlPFC to attenuate nociceptive processing, suggesting that nostalgic analgesia operates through the thalamus-PAG-dlPFC pathways.

The key independent variable we focused on in the current study, nostalgia, is a complicated emotion (Hepper et al., 2014). To balance individual differences between subjects, we adopted a within-participant design to explore the analgesic effect of nostalgia. However, it is possible that participants could be distracted by the control images in the subsequent trial if they continued to feel the effects of their nostalgic immersion status induced by the images they saw in the earlier trial. In this case, the nostalgic effect would have shrunk visibly, although it is noteworthy that we did observe a tangible impact of nostalgia on pain relief. Similarly, using a between-participant design and much stronger nostalgic materials would be better used in future studies to achieve a stronger and more stable nostalgic status and to examine the best strategies to operationalize psychological analgesia. Another limitation is that we only examined participants within a limited age range. It is essential to investigate whether the analgesic effect changes with age to consider its potential clinical applications.

In conclusion, the current study results reveal that the thalamus, as a critical brain region for pain modulation, is also related to the analgesic effect associated with nostalgia. Meanwhile, thalamus-PAG connectivity in the cue stage and PAG-dlPFC connectivity in the pain stage also suggest potential analgesic pathways. These findings offer implications and perspectives for the further development and improvement of nondrug, psychological analgesia.
